# The complete mitogenome sequence of the coral lily (*Lilium pumilum*) and the Lanzhou lily (*Lilium davidii*) in China

**DOI:** 10.1515/biol-2020-0102

**Published:** 2020-12-31

**Authors:** Xiangying Qi, Kaiqi Wang, Liping Yang, Zhenshan Deng, Zhihong Sun

**Affiliations:** China Lily Laboratory, Shaanxi Engineering and Technological Research Center for Conversation and Utilization of Regional Biological Resources, Yan’an University, Yan’an, 716000, Shaanxi, China; School of Advanced Agriculture and Bioengineering, Yangtze Normal University, Chongqing, 408100, China

**Keywords:** complete mitogenome sequence, *Lilium* species, Illumina sequencing, phylogenetic tree, variable sites

## Abstract

**Background:**

The mitogenomes of higher plants are conserved. This study was performed to complete the mitogenome of two China *Lilium* species (*Lilium pumilum* Redouté and *Lilium davidii* var. *unicolor* (Hoog) cotton).

**Methods:**

Genomic DNA was separately extracted from the leaves of *L. pumilum* and *L. davidii* in triplicate and used for sequencing. The mitogenome of *Allium cepa* was used as a reference. Genome assembly, annotation and phylogenetic tree were analyzed.

**Results:**

The mitogenome of *L. pumilum* and *L. davidii* was 988,986 bp and 924,401 bp in length, respectively. There were 22 core protein-coding genes (including *atp1, atp4, atp6, atp9, ccmB, ccmC, ccmFc, ccmFN1, ccmFN2, cob, cox3, matR, mttB, nad1, nad2, nad3, nad4, nad4L, nad5, nad6, nad7 and nad9*), one open reading frame and one ribosomal protein-coding gene (*rps12*) in the mitogenomes. Compared with the *A. cepa* mitogenome, the coding sequence of the 24 genes and intergenic spacers in *L. pumilum* and *L. davidii* mitogenome contained 1,621 and 1,617 variable sites, respectively. In the phylogenetic tree, *L. pumilum* and *L. davidii* were distinct from *A. cepa* (NC_030100).

**Conclusions:**

*L. pumilum* and *L. davidii* mitogenomes have far distances from other plants. This study provided additional information on the species resources of China *Lilium*.

## 1 Introduction

The mitogenomes of higher plants are usually highly conserved and highly variable across the plant taxa [1]. Mitogenome is usually transmitted to the progeny from the maternal parent [2]. Mitogenome of higher plants ranges from 200 to 2,400 kb due to the expansion and the reduction of the intergenic region [3,4]. The constant recombination and great variation in the spacer regions in mitochondrial DNA (mtDNA) promote the evolution of higher plants. During evolution, most of the genes in plant mtDNA have been lost [5]. Compared to the chloroplast, the plant mitogenome has lower mutation rates [6]. Accordingly, the mitogenome plays a specific role in the evolution of plants. There are many studies sequencing the complete mitogenomes of higher plants and describing their genome features [3].

The genus *Lilium* (Liliaceae) is a very important economic plant worldwide [7,8]. *Lilium* species are perennial herbaceous plants with ornamental and notable medicinal value [9]. In the research and development of traditional Chinese medicine (TCM), the pharmaceutical characteristics of *Lilium* species, especially the extracts including polysaccharides, kaempferol and jatropham, have been widely studied [10,11]. For instance, the extract from *Lilium* species showed antidiabetic, antioxidant, antiapoptotic and health-protective properties [10,12,13]. These properties promoted the research and the exploration of bioactive and potential pharmaceutical activities in *Lilium* in the TCM field.

The genus *Lilium* is composed of approximately 115 species [8,14], and about 55 species are centrally distributed in China [15]. Some of the *Lilium* species in China have not been well identified yet. The coral lily *Lilium pumilum* Redouté and the Lanzhou lily *Lilium davidii* var. *unicolor* (Hoog) cotton are two Chinese species with ornamental and economic values [16,17]. Both *L. pumilum* and *L. davidii* are the important original materials of TCM [16,18]. *L. pumilum*, which has beautiful bright red flowers, is an endangered species in China [19]. However, the genetic evolution of these two species has not been reported until now.

This study was performed to examine the complete mitogenome of the two *Lilium* species. The mitogenomes of *L. pumilum* and *L. davidii* were sequenced using Illumina HiSeq X Ten sequencing platform. The variations in the mitogenomes were identified by referring to the reference mitogenome sequence of the closely related *Allium cepa* (GenBank: KU318712.1, NC_030100.1). The phylogenetic tree analysis was performed to identify the evolution distance of these two species from other plants with completed mitogenome sequences. This study will enrich the Chinese germplasm bank of lily.

## 2 Materials and methods

### 2.1 Plant materials and sequencing

The leaf samples of *L. pumilum* and *L. davidii* were obtained from Shaanxi Engineering and Technological Research Center for Conversation and Utilization of Regional Biological Resources, Yan’an, China. Both species were wild type and uncultivated plant genetic resources in China. Leaves were immersed into liquid nitrogen and stored at −80°C before use. Triplicate genomic DNA samples were extracted from leaves of each species using the cetyltrimethylammonium bromide (CTAB) method [20].

Following the genomic DNA quantity measurement (Qubit 3.0; Invitrogen, USA), samples were subjected to fragmentation, library construction (TruSeq™ DNA Sample Prep Kit; Illumina Inc, USA), PCR amplification and sequencing on the platform of Illumina HiSeq X Ten (pair-end 2 × 150 bp). Single-cell sequencing was performed on the PacBio RSII platform with 8–10 kb fragments.

### 2.2 Genome assembly and annotation

The Illumina raw data were processed using FastQC (http://www.bioinformatics.babraham.ac.uk/projects/fastqc/), followed by alignment with the PacBio RS sequencing reads using BlasR [21] and data assembly using SPAdes-3.10.1 program [22]. Long sequences assembled (>500 bp) with high alignment ratio were considered as candidates. Mt contigs were screened from these assembled candidates by blasting against the plant mitogenomes in NCBI Organelle Genome Resources (https://www.ncbi.nlm.nih.gov/genome/organelle/). The Illumina clean data were then compared back to the assembled mt contigs, followed by modification (bug fix) using GapCloser (v1.12; http://soap.genomics.org.cn/soapdenovo.html). The mitogenomic data of the two species sequenced were finally obtained.

The tRNA sequences in the obtained mitogenomes were recognized using tRNAscan-SE (http://lowelab.ucsc.edu/tRNAscan-SE/). The protein-coding genes and rRNA-coding genes in the mitogenomes were identified by aligning against the closely related *A. cepa* mitogenome sequence (GenBank: KU318712.1; NC_030100), with the threshold of alignment *p* < e value 1e^−5^. The exon and intron regions of genes were manually checked, and the gene sets in the mitogenome were identified. The variable sites in the coding sequence (CDS) of protein-coding genes and rRNA genes and intergenic spacers, as well as the structural variations (>100 bp) in the complete mitogenome sequence of the two *Lilium* species, were identified by comparison with *A. cepa* mitogenome (NC_030100) using the MUMmer and LASTZ alignment tools with default parameters.

### 2.3 Phylogenetic tree construction

The phylogenetic tree was constructed using the amino acid sequences of 29 plants with the available complete mitogenome sequences, including *A. cepa* (NC_030100.1), *Bambusa oldhamii* (EU365401.1), *Lolium perenne* (JX999996.1), *Zea mays* (DQ645536.1), *Triticum aestivum* (GU985444.1), *Oryza sativa* (NC_011033.1), *Vicia faba* (KC189947.1), *Platycodon grandiflorus* (MG775429.1), *Nicotiana tabacum* (NC_006581.1), *Daucus carota* subsp. *Sativus* (NC_017855.1), *Arabidopsis thaliana* (NC_037304.1), *Gossypium harknessii* (JX536494.1), *Olea europaea* (MG372117.1), *Spirodela polyrhiza* (NC_017840.1), *Coleochaete scutata* (MN613583.1), *Chlorella vulgaris* (MK948101.1), *Hyoscyamus niger* (NC_026515.1), *Eleusine indica* (NC_040989.1), *Physochlaina orientalis* (NC_044153.1), *Sorghum bicolor* (NC_008360.1), *Cocos nucifera* (NC_031696.1), *Salix purpurea* (NC_029693.1), *Brassica juncea* (NC_016123.1), *Panax ginseng* (KF735063.1), *Cynodon dactylon* x *Cynodon transvaalensis* (MK175054.1), *Solanum lycopersicum* (MF034192.1), *Glycine max* (NC_020455.1) and the two *Lilium* species (*L. pumilum* and *L. davidii*). The complete mitogenome sequences of these species were aligned using clustalW2 (https://www.ebi.ac.uk/Tools/msa/clustalw2/) with default parameters and manual check. The mitogenome-wide phylogenetic tree was subsequently constructed based on the concatenated amino acid sequences of 23 protein-coding genes and visualized using MEGA 5.2 Software [23] (maximum-likelihood method, *n* = 1,000 times, bootstrap algorithm).

## 3 Results and discussion

### 3.1 General features of the two mitogenomes

The Illumina sequencing generated 36,455,269 clean reads in *L. pumilum* and 59,258,050 clean reads in *L. davidii* with an averaged GC content of 45.06 and 44.87%, respectively. Only contigs >500 bp were included for the annotation of mitogenome. Accordingly, the mitogenome of *L. pumilum* was 988,986 bp in length with 45.06% GC content, and the mitogenome of *L. davidii* was 924,401 bp in length with 44.87% GC content (Table 1). These genomes were longer than 773,279 bp in *Vitis vinifer*a [5], 490,520 bp in *O. sativa* [24], 316,363 bp in *A. cepa* [25], 678,653 bp in *C. nucifera* [26] and 452,526 bp in *T. aestivum* cv. Chinese Yumai [27], and smaller than 1,106,521 bp in *Schisandra sphenanthera* (Austrobaileyales) [28]. The obtained mitogenomes (988,986 and 924,401 bp) were of median size compared to these plants, but were relatively larger than the aforementioned monocotyledons. This difference might due to the expansion of the intergenic region [27,29]. The annotation of variable sites in the mitogenome would be of great importance.

**Table 1:** General features of the assembled contigs (>500 bp) from mitogenome sequencing in *Lilium pumilum* Redouté and *Lilium davidii* var. *unicolor* (Hoog) cotton

Species	*L. pumilum*	*L. davidii*
Total number	17	17
Total length (bp)	988,986	924,401
Average length (bp)	58,475.65	54,376.53
N50 length (bp)	75,221	75,303
N90 length (bp)	30,276	36,946
Maximum length (bp)	256,615	159,127
Minimum length (bp)	20,912	1,933
GC content (%)	45.06	44.87
No. of variable sites	1,621	1,617
No. of variable sites in CDS (*n* (%))	882 (54.41)	881 (54.48)
No. of variable sites in the intergenic spacers (*n* (%))	739 (45.59)	736 (45.52)
Length of CDS and intergenic spacers (%)	42.23	45.18

N50 and N90, the length for which the collection of all contigs of that length or longer contains at least 50 and 90% of the total of the lengths of the contigs, respectively.

### 3.2 Annotation of variable sites in the mitogenomes

There were 1,621 and 1,617 variable sites in the CDS of the 23 annotated genes and the intergenic spacers between genes in the mitogenome of *L. pumilum* and *L. davidii* in comparison to *A. cepa* mitogenome, respectively (Table 1). The number of indels in the complete mitogenome sequence of *L. pumilum* (*n* = 45) was higher than that of *L. davidii* (*n* = 34). In addition, the variable sites in the mitogenomes in *L. pumilum* and *L. davidii* aligned onto 22 core protein-coding genes (including *atp1, atp4, atp6, atp9, ccmB, ccmC, ccmFc, ccmFN1, ccmFN2, cob, cox3, matR, mttB, nad1, nad2, nad3, nad4, nad4L, nad5, nad6, nad7 and nad9*), one open reading frame (ORF; *orf725*) and one ribosomal protein-coding gene (*rps12*) in *A. cepa* (Table 2). There were 733 and 484 variable sites in gene CDS and intergenic spacers in *L. pumilum* and *L. davidii*, respectively (Table 2). tRNA genes were not identified. The number of annotated protein-coding genes in the complete mitogenome sequence of *L. pumilum* and *L. davidii* was significantly lower than that in other reported plants, including 35 genes in *T. aestivum* cv. Chinese Yumai [27], 37 genes in *V. vinifer*a [5], 41 genes in *S. sphenanthera* [28] and 72 genes in *C. nucifera* L. (coconut palm) [26], but was identical to the 23 protein-coding genes in the mitogenome of *A. cepa* [25]. Our previous research showed that the mitochondrial genome size of different plants is very different from others and the reference mitogenome of *A. cepa* due to the expansion and reduction of the intergenic region [27,29]. The narrowed gene number in *L. pumilum* and *L. davidii* might be due to the small number of known genes in the reference mitogenome in *A. cepa*. However, the larger mitogenome size in *L. pumilum* and *L. davidii* compared with the reference mitogenome of *A. cepa* might suggest that there was a large percent of mitogenomic regions with a number of unknown genes, which might be of great values for the function and evolution analysis of both *L. pumilum* and *L. davidii* species.

**Table 2:** Summary of the variable sites in the CDS of the 24 annotated genes and the intergenic spacers in the mitogenomes of *Lilium pumilum* Redouté and *Lilium davidii* var. *unicolor* (Hoog) cotton in comparison to *Allium cepa* mitogenome (NC_030100)

Gene_ID	Number	CDS			Strand	Number	Intergenic spacers		Strand
		Syn	Start	End			Start	End	
atp1	110	70	51,272	52,795	+	96	51,272	52,795	+
atp4	30	9	246,733	247,296	+	12	246,733	247,296	+
atp6	0	0	—	—	—	35	82,415	83,221	+
atp9	17	15	103,573	103,842	+	0	—	—	—
ccmB	31	13	278,555	279,175	+	5	278,555	279,175	+
ccmC	0	0	—	—	—	40	213,897	214,712	+
ccmFc	39	15	67,963	68,717	−	5	66,290	68,717	−
ccmFN1	60	14	148,328	149,374	+	38	148,328	149,374	+
ccmFN2	28	10	287,429	288,037	−	10	287,429	288,037	−
cob	26	7	301,112	302,302	−	2	301,112	302,302	−
cox3	0	0	—	—	—	11	238,167	238,976	−
matR	89	34	64,084	66,084	−	8	64,084	66,084	−
mttB	40	15	134,707	135,456	+	0	—	—	—
nad1	8	5	63,268	63,525	−	11	12,513	63,525	−
nad2	14	2	31,139	31,326	−	6	91,121	31,326	+
nad3	8	1	18,764	19,120	+	0	—	—	—
nad4	7	3	168,424	168,885	−	20	159,945	168,885	−
nad4L	10	9	246,284	246,586	+	13	246,284	246,586	+
nad5	52	12	1,082	2,302	−	42	1,082	290,301	−
nad6	14	7	180,834	181,802	−	70	180,834	181,802	−
nad7	3	2	28,132	28,275	−	13	21,417	28,275	−
nad9	23	11	205,033	205,530	−	40	205,033	205,530	−
orf725	101	75	75,188	77,365	+	1	75,188	77,365	+
rps12	23	15	19,166	19,543	+	6	19,166	19,543	+
Total	733	334				484			

Syn, synonymous.

Of the protein-coding genes, *atp1*, *matR* and *ccmFN1* had the highest number of variable sites (110, 89 and 52, respectively) in the CDS regions in reference to *A. cepa* (Table 2). In addition, 96, 70, 42, 40 and 40 variable sites were identified in the intergenic spacers around *atp1*, *nad6*, *nad5*, *ccmC* and *nad9*, respectively. These results might reveal that these genes are of great value for the evolution of *L. pumilum* and *L. davidii* species [2]. By contrast, a small number of variable sites were found in the CDS of *nad1*, *nad3*, *nad7* and *cox3*, and in the intergenic spacers around them (Table 2). These results might suggest their smaller contribution to the evolution of the two mitogenomes. The fact that there were no variable sites in the CDS of *cox3*, *atp9* and *nad3* genes showed that the CDS regions are well-conserved regions between the two species sequenced and *A. cepa* [30]. In addition, this conservation might govern the conservative evolution of mitogenome during the evolution of these genes [31].

### 3.3 Structural variations in the mitogenomes

The identified structural variations in the mitogenomes of *L. pumilum* and *L. davidii* are listed in Table 3. There were 14 variations in both mitogenomes with a length range of 100–401 bp. Most of the variations were insertion type (42.86%, 6/14), followed by deletion (35.71%, 5/14) and complex indel (21.43%, 3/14; Table 3). All the variations in *L. pumilum* were circular types, and half variations in *L. davidii* were circular types. One linear structural variation happened in *ccmFc*, *atp1* and *nad1* genes, which had a large number of variable sites (Tables 2 and 3). These variations might be of important values in the evolutionary history of *L. pumilum* and *L. davidii* [32,33].

**Table 3:** Summary of the total structural variations (SVs) in the mitogenome of *Lilium pumilum* Redouté and *Lilium davidii* var. *unicolor* (Hoog) cotton*. L. davidii* in comparison to *Allium cepa* mitogenome (NC_030100)

Target sequence (TS)			Query sequence (QS)			Strand	SV type	L. in TS	L. in QS	Block type	Block number
*L. pumilum*	Start	End	*L. davidii*	Start	End						
Circle1	30,927	38,044	Circle12	4,373	5,244	+	ComplexIndel	7,118	872	400	A4-4
Circle1	47,494	47,494	Circle12	14,695	16,655	+	Insertion	0	1,961	100	4–4.5
Circle1	91,405	91,685	Linear1	113,819	113,819	+	Deletion	281	0	401	A11–12
Circle1	94,577	94,826	Linear1	116,766	116,766	+	Deletion	250	0	101	11–12.13
Circle1	217,901	217,901	Circle8	13,072	14,014	+	Insertion	0	943	101	21–23.24
Circle1	218,291	218,291	Circle8	14,405	14,942	+	Insertion	0	538	101	21–24.25
Circle1	244,299	244,736	Linear1	0	0	+	Deletion	438	0	401	A26–30
Circle1	253,016	253,016	Linear1	8,277	14,259	+	Insertion	0	5,983	101	26–30.31
Circle12	4,711	4,711	Linear1	37,830	38,053	—	Insertion	0	224	111	32–37.38
Circle3	8,779	13,019	Circle1	8,787	8,787	+	Deletion	4,241	0	101	38–44.45
Circle5	521	2,507	Circle11	10,164	12,818	+	ComplexIndel	1,987	2,655	101	41–48.49
Circle6	33,521	36,804	Circle9	21,210	21,210	+	Deletion	3,284	0	100	48–56.57
Circle8	5,820	24,748	Linear2	46,265	71,977	+	ComplexIndel	18,929	25,713	101	54–63.64
Circle9	4,579	4,579	Linear3	4,457	5,644	+	Insertion	0	1,188	101	56–66.67

Target and query length, the length of SV in the target and query sequences; L. in TS, length of corresponding SV in target sequence; L. in QS, length of corresponding SV in query sequence.

### 3.4 Evolutionary analysis of the two *Lilium* species

**Figure 1:**
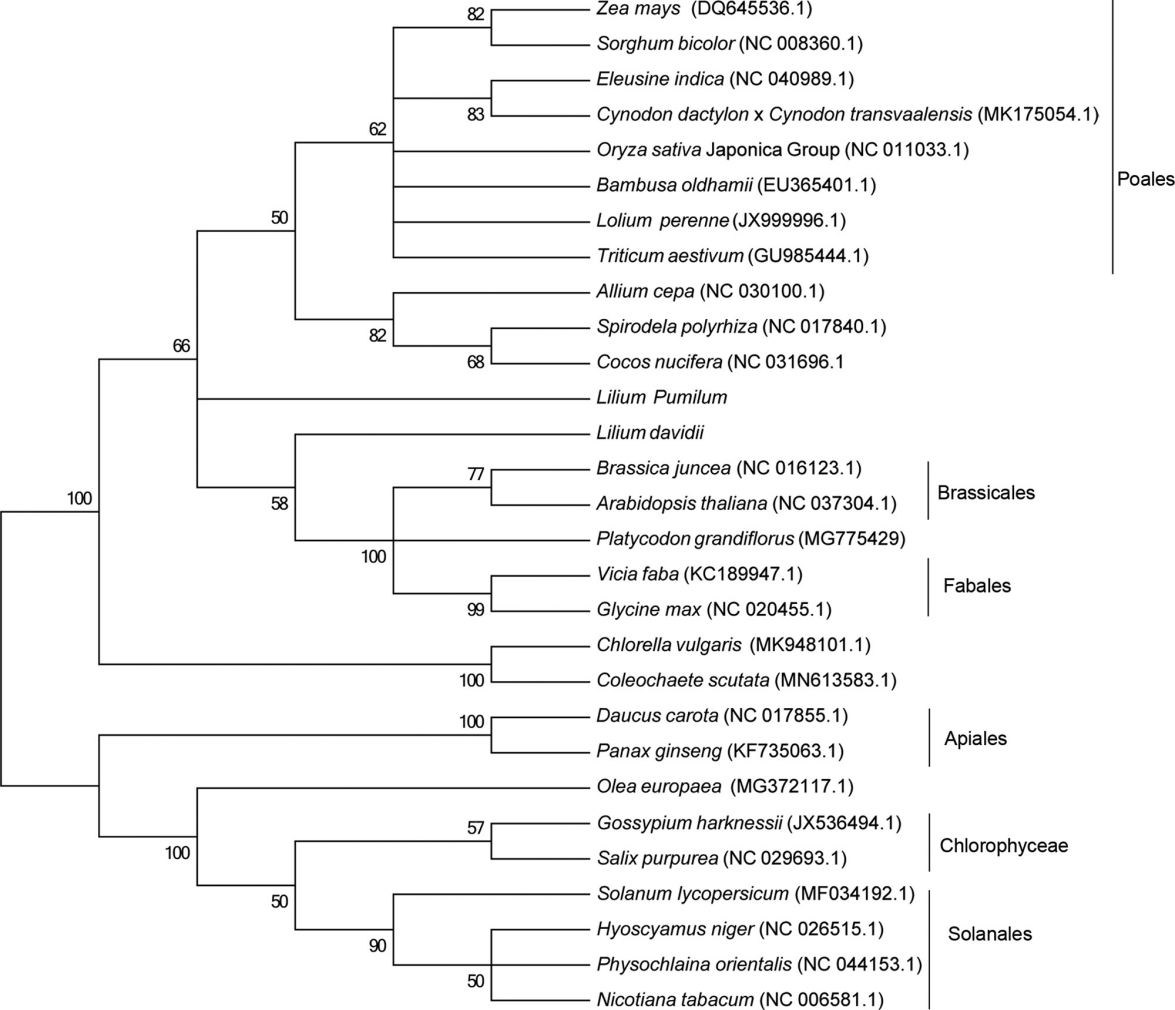
The phylogenetic tree inferring from the concatenated amino acid sequences of 23 protein-coding genes of the 29 plants. The amino acid sequences of 23 protein-coding genes in the complete mitogenome sequences of other 27 plants were retrieved from the NCBI (https://www.ncbi.nlm.nih.gov/nuccore).

The phylogenetic tree was constructed based on the concatenated amino acid sequences of the 23 protein-coding genes in the two *Lilium* species and other 27 plants. Figure 1 shows the phylogenetic evolutionary position of *L. pumilum* and *L. davidii*. The two *Lilium* species had close relationships with the Brassicales and Poales clades, while *A. cepa* showed close relationships with Poales clades and *C. nucifera* (Figure 1). Both *L. pumilum* and *L. davidii* were distant from *A. cepa* (NC_030100; Figure 1). These distinct genetic distances between *L. pumilum* or *L. davidii* and the reference mitogenome of *A. cepa* might be due to a high number of variable sites in the mitogenomes of *L. pumilum* and *L. davidii* species.

A previous phylogenetic study based on 79 chloroplast genes showed that *L. longiflorum* (Liliaceae) and *Alstroemeria aurea* Graham (Alstroemeriaceae) were clustered into the Asparagales + Commelinids clade, showing the close genetic relationships between them [34]. İkinci et al. [35] reported that there were remote evolutionary distances among different lily species from different regions in European and other countries based on the internal transcribed spacer regions of nuclear ribosomal DNA. Similar results of Japan lily species were reported by Nishikawa et al. [36]. We suppose that the more number of the annotated protein-coding genes in the mitogenome of the two lily species, the more detailed information about lily’s evolution analysis will be obtained.

## 4 Conclusions

In summary, we obtained the complete mitogenome sequences of *L. pumilum* (988,986 bp) and *L. davidii* (924,401 bp) and presented the phylogenetic relationships of them for the first time. A total of 24 protein-coding genes, including one ribosomal protein-coding gene (*rps12*), were annotated by aligning with the reference genome in *A. cepa* (GenBank: KU318712.1; NC_030100). The phylogenetic tree analysis showed that these two species had distinct mitochondrial genomes from other plants including *A. cepa*. The large unannotated sections in the mitogenome (988,986 or 924,401 bp) in *Lilium* species compared with that in the *A. cepa* reference genome (316,363 bp) should be performed in the further research.
